# Design and Finite Element Simulation of a Novel 3D-CMUT Device for Simultaneous Sensing of In-Plane and Out-of-Plane Displacements of Ultrasonic Guided Waves

**DOI:** 10.3390/s23218706

**Published:** 2023-10-25

**Authors:** Sai Zhang, Wei Lu, Ailing Wang, Guodong Hao, Renxing Wang, Mehmet Yilmaz

**Affiliations:** 1 Institute of Ultrasonic Testing, Jiangsu University, Zhenjiang 212013, China; 2 State Key Laboratory of Dynamic Testing Technology, North University of China, Taiyuan 030051, Chinawangrenxin@nuc.edu.cn (R.W.); 3 Institute of Materials Science and Nanotechnology, Bilkent University, Ankara 06800, Turkey; 4 UNAM—National Nanotechnology Research Center, Bilkent University, Ankara 06800, Turkey

**Keywords:** ultrasonic guided waves, in-plane displacement, out-of-plane displacement, CMUT, 3D

## Abstract

In this study, we introduce a physical model of a three-dimensional (3D) guided wave sensor called 3D-CMUT, which is based on capacitive micro-machined ultrasonic transducers (CMUTs). This 3D-CMUT sensor is designed to effectively and simultaneously obtain 3D vibration information about ultrasonic guided waves in the out-of-plane (*z*-direction) and in-plane (*x* and *y*-directions). The basic unit of the 3D-CMUT is much smaller than the wavelength of the guided waves and consists of two orthogonal comb-like CMUT cells and one piston-type CMUT cell. These cells are used to sense displacement signals in the *x*, *y*, and *z*-directions. To ensure proper functioning of the 3D-CMUT unit, the resonant frequencies of the three composed cells are set to be identical by adjusting the microstructural parameters appropriately. Moreover, the same sensitivity in the *x*, *y*, and *z*-directions is theoretically achieved by tuning the amplification parameters in the external circuit. We establish a transient analysis model of the 3D-CMUT using COMSOL finite element simulation software to confirm its ability to sense multimode ultrasonic guided waves, including A_0_, S_0_, and SH_0_ modes. Additionally, we simulate the ball drop impact acoustic emission signal on a plate to demonstrate that the 3D-CMUT can not only utilize in-plane information for positioning but also out-of-plane information. The proposed 3D-CMUT holds significant potential for applications in the field of structural health monitoring (SHM).

## 1. Introduction

Recently, ultrasonic guided waves, including Lamb waves and shear horizontal waves, have gained significant attention for damage-detection techniques in plate-like structures for non-destructive inspection and structural health monitoring (SHM) [1,2,3]. These guided waves offer the unique capability of long-range and through-the-thickness interrogation of structures. Different guided wave modes, such as the lowest order asymmetric Lamb wave (A_0_ mode), symmetric Lamb wave (S_0_ mode), and shear horizontal wave (SH_0_ mode), exhibit distinct displacement characteristics on the surface of a plate. The A_0_ and S_0_ modes have dominant out-of-plane and in-plane displacements, respectively, while the SH_0_ mode only exhibits in-plane displacement [4]. However, when these guided waves interact with different types of defects, mode conversion and scattering may occur, leading to a complex wave field with various wave modes [5]. Therefore, the effective acquisition of both in-plane and out-of-plane displacements of guided waves is crucial for guided wave field analysis and damage imaging. It has been demonstrated that obtaining 3D information about particle displacements is essential for accurate damage imaging and evaluation [6].

To date, a number of sensors have been used to capture ultrasonic guided waves in structures. Some of these sensors include piezoelectric sensors based on the piezoelectric effect [7], optical fiber grating sensors based on the grating diffraction effect [8], and electromagnetic acoustic transducers based on the magnetostrictive effect [9]. Although those sensors have a good performance in practice, in areas such as broad bandwidth and high sensitivity, they can only obtain either in-plane or out-of-plane displacement information concerning guided waves. Namely, none of these guided wave sensors can simultaneously obtain three-dimensional information about guided waves at a small measurement point, which significantly limits the development of ultrasonic guided wave damage-detection techniques. To the best of our knowledge, the only effective measurement method that can acquire in-plane and out-of-plane displacements of guided waves at a single point or a small area is the three-axis scanning laser Doppler vibrometer (3D-SLDV) [10]. However, the 3D-SLDV is an expensive and bulky measurement instrument, which may not be able to replace sensors for real-time SHM.

Recently, capacitive micro-machined ultrasonic transducers (CMUTs) have emerged as a new and promising type of ultrasonic transducer [11]. CMUTs offer numerous advantages, such as matured fabrication-processing approaches, higher bandwidth, and better integration. Various important applications based on CMUTs, including medical imaging and high-intensity focused ultrasound (HIFU) therapy, have been investigated [12]. More recently, researchers have started to design and fabricate CMUT-based sensors for structural health monitoring (SHM) applications. In the literature, by exploiting the conduction principle of capacitance changes, CMUT-based sensors for an acoustic emission (AE) application have been designed [13,14,15,16,17,18]. In 2006, D. Ozevin et al. developed CMUT-based AE sensors that span the frequency range 100–500 kHz [13]. Although they have some response to out-of-plane displacements, they are 50 times less sensitive than conventional piezoelectric emission sensors. H. Saboonchi et al. used the MetalMUMPs technique to design and realize an out-of-plane-sensing MEMS AE transducer with high-aspect ratio geometry, which has a sensitivity comparable to piezoelectric transducers [14]. In order to realize in-plane-displacement sensing, M. Kabir et al. designed, characterized, and compared in-plane-sensing interdigital CMUTs with area changes and gap changes from both theoretical and simulation aspects [15]. Recently, W. Lu and co-authors demonstrated the feasibility of sensing A_0_ and S_0_ Lamb waves using an out-of-plane-sensitive resonant CMUT [16], and they also performed an in-plane sensing analysis of comb-like CMUTs using an analytical small-signal model [17]. To our knowledge, CMUT sensors have received more attention in the SHM field, but they also face challenges in bandwidth, sensitivity, signal-to-noise ratio, and the reliability of the preparation process, etc [18].

In this paper, a physical model of a CMUT-based ultrasonic guided wave sensor (3D-CMUT) that is capable of detecting three-dimensional information is proposed. This 3D-CMUT is designed to effectively and simultaneously obtain 3D vibration information about ultrasonic guided waves in the out-of-plane (*z*-direction) and in-plane (*x* and *y*-directions) directions. We further establish the frequency and transient analysis models of the 3D-CMUT using COMSOL (version 6.1) finite element simulation software to confirm its ability to sense multimode ultrasonic guided waves, including A_0_, S_0_, and SH_0_ modes. Additionally, we simulate the ball drop impact acoustic emission signal on a plate to demonstrate that the 3D-CMUT does not only utilize in-plane information for positioning but also utilizes out-of-plane information. Our numerical study suggests that the proposed 3D-CMUT can simultaneously sense in-plane and out-of-plane displacements of ultrasonic guided waves with multiple modes, and that it is also useful for acoustic emission-sensing in a plate and thus has promising applications in the field of SHM. Our numerical studies and novel design will facilitate the fabrication of novel 3D-CMUT devices.

## 2. Design of 3D-CMUT

### 2.1. Model and General Design Method

**Figure 1:**
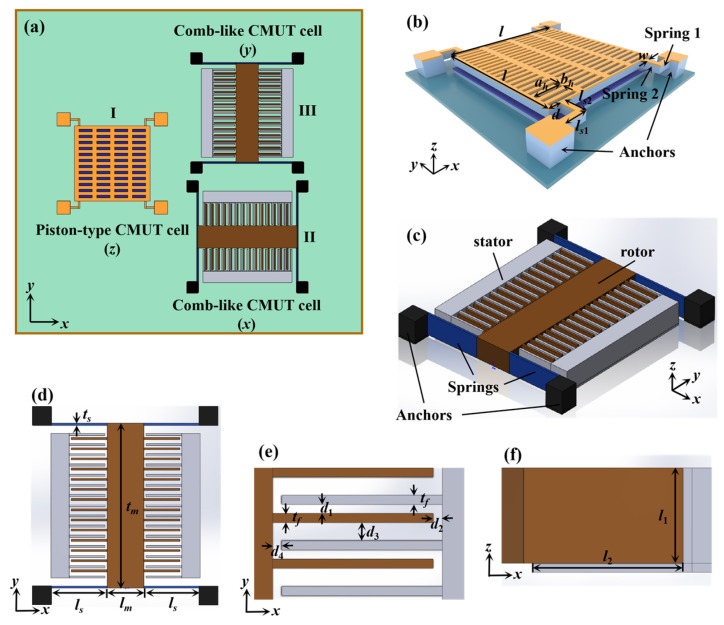
Schematic diagram of the proposed 3D-CMUT. (**a**) One unit of the 3D-CMUT; (**b**) piston-type CMUT cell; (**c**) comb-like CMUT cell; (**d**) vertical (top-down) view of the comb-like CMUT cell; (**e**,**f**) show the partial enlarged views of crossed fingers in (**d**) in the *x*-*y* plane and the *x*-*z* plane, respectively.

The proposed 3D-CMUT device consists of two orthogonal comb-like CMUT cells and one piston-type CMUT cell, which are designed to sense displacement signals in the *x*, *y* and *z*-directions, respectively. Hereby, a 3D-CMUT device is designed as shown in Figure 1. The proposed 3D-CMUT device is divided into three sensing regions, i.e., one out-of-plane-sensing region and two in-plane-sensing regions, to sense ultrasonic guided waves in the *z*, *x*, and *y*-directions. As shown in Figure 1a, one unit of our proposed 3D-CMUT includes a piston-type CMUT cell (labeled as I), for sensing out-of-plane displacements (*z*-direction), and two orthogonally placed comb-like CMUT cells (labeled as II and III), for sensing in-plane displacements (*x* and *y*-directions). The piston-type CMUT cell and the comb-like CMUT cell were theoretically and numerically studied in our previous work in refs. [16] and [17], respectively.

The piston-type CMUT cell, as shown in Figure 1b, is designed to be sensitive to out-of-plane displacements. The piston-type CMUT cell is composed of an electroplated layer (Au, height *d_Au_*), top electrode (polycrystalline silicon, height *d_top_*), gap (air, height *d_gap_*), insulation layer (Si_3_N_4_, height $d_{Si_{3}N_{4}}$), bottom electrode (polycrystalline silicon, height *d_bottom_*), springs (height *d_Au_* + *d_top_*) and anchors, where the L-shaped springs and the anchors serve to keep the top electrode suspended. The lengths of each layer along the *x*-axis and the *y*-axis are set as *l*. The L-shaped springs are divided into two sections with the widths being *w*, where the first section [see Figure 1b, Spring 1] has the length of *l_s_*_1_ and the second section [see Figure 1b, Spring 2] has the length of *l_s_*_2_-*w*. The distances between the springs and edges are set as *d*. The lengths of through-holes along the *x*-axis and the *y*-axis are labeled as *a_h_* and *b_h_*, respectively.

The comb-like CMUT cell, as shown in Figure 1c, is designed to be sensitive to in-plane displacements. It is composed of four anchors, four springs, two stators that are electrically connected to each other, and a rotor. The anchors are represented by black regions, the springs by dark blue regions, the stators by gray regions, and the rotor by brown regions. The rotor consists of a proof mass and multiple comb fingers. The material used for the comb-like CMUT cell is conductive polycrystalline silicon. The stators and the rotor act as the mechanically fixed electrodes and the movable electrode, respectively. The outstretched fingers of the rotor and the stators form a variable interdigital capacitor. The anchors and springs work together to hold the suspended rotor in place. As shown in the vertical view of the cell [Figure 1d], the width of springs, the length of springs, the width of the proof mass, and the length of the proof mass are denoted by *t*_s_, *l_s_*, *t_m_*, and *l_m_*, respectively. The interdigital capacitor can be divided into four different parallel-plate capacitors according to the air gap [Figure 1e], where the widths of these air gaps are represented by *d*_1_, *d*_2_, *d*_3_, and *d*_4_, respectively. The widths of the outstretched fingers are set as *t_f_*. In the *x-z* plane [Figure 1f], the height and the length of the facing area between adjacent fingers are represented by *l*_1_, and *l*_2_, respectively.

The sensitivity *H_z_* (*ω*) of the piston-type CMUT cell, and *H*_*x*,*y*_ (*ω*) of the comb-like CMUT cell are defined as the ratio of the output voltage to the input speed in the unit of dB(@V·s/m)

(1) $$H_{z}(\omega )=20\log_{10}\left|-\frac{iR_{z}U_{z}\varepsilon_{0}V_{dc}SQ\omega^{3}}{i\omega {\left(a-d_{0}\right)}^{2}[Q(\omega_{0,z}^{2}-\omega^{2})+i\omega \omega_{0,z}]}\right|$$

(2) $$H_{x,y}(\omega )=20\log_{10}\left|\frac{n\varepsilon_{0}\varepsilon l_{1}l_{2}V_{dc}R_{x,y}U_{x,y}Q\omega^{2}\left(\frac{1}{{\left(d_{1}-y_{b}\right)}^{2}}-\frac{1}{{\left(2d_{1}+y_{b}\right)}^{2}}\right)}{Q(\omega_{0,x,y}^{2}-\omega^{2})+i\omega \omega_{0,x,y}}\right|$$

where,

$$a=\frac{d_{Si_{3}N_{4}}}{\varepsilon_{Si_{3}N_{4}}}+d_{gap}$$

$$d_{0}=\frac{V_{dc}^{2}\varepsilon_{0}Sa}{2(ka^{3}-V_{dc}^{2}\varepsilon_{0}S)}$$

$$y_{b}=\frac{3\varepsilon_{0}nd_{1}l_{1}l_{2}V_{dc}^{2}}{8kd_{1}^{3}-9\varepsilon_{0}nl_{1}l_{2}V_{dc}^{2}}$$

and *k* is the effective spring constant, *S* is the electrode facing area of a piston-type CMUT cell, *n* is the number of fingers outstretched from the rotor, *V_dc_* is the DC bias voltage applied on the electrodes, *ε*_0_ is the dielectric constant of vacuum, $\varepsilon_{Si_{3}N_{4}}$ is the relative permittivity of Si_3_N_4_ and *Q* is the quality factor of the CMUT cell. *ω*_0,*z*_ and *ω*_0,*x*,*y*_ represent the first natural angular frequency of the piston-type CMUT cell and the orthogonal comb-like CMUT cell, respectively.

In order to make the 3D-CMUT truly reflect the out-of-plane displacements and the in-plane displacements of the arrived guided waves, the 3D-CMUT needs to be carefully designed to meet two basic principles. Namely, the consistency in the sensitive frequency band and the consistency in the average sensitivity, where the former refers to the sensitive frequency band of the out-of-plane-sensing region (and the in-plane-sensing regions should be exactly the same), and the latter refers to the average sensitivity in the sensitive frequency band of the out-of-plane-sensing region (and the in-plane-sensing regions should also be the same). For a narrowband 3D-CMUT, the consistency in the sensitive frequency band can be realized by designing a piston-type CMUT cell with a resonant frequency and quality factor that is the same as the comb-like CMUT cell; for a broadband 3D-CMUT, the consistency in the sensitive frequency band can be realized by using a genetic algorithm, as reported in Ref. [16]. Whether it is a narrowband 3D-CMUT or broadband 3D-CMUT, the average sensitivity of each sensing region, in the consistent sensitive angular frequency band of [*ω*_1_, *ω_N_*], can be expressed as:

(3) $${\bar{H}}_{z}(\omega_{1}:\omega_{N_{z}})=20\log_{10}\left(\frac{R_{z}U_{z}{\bar{i}}_{total,z}(\omega_{1}:\omega_{N_{z}})}{{\bar{v}}_{z}(\omega_{1}:\omega_{N_{z}})}\right)$$

(4) $${\bar{H}}_{x,y}(\omega_{1}:\omega_{N_{x,y}})=20\log_{10}\left(\frac{R_{x,y}U_{x,y}{\bar{i}}_{total,x,y}(\omega_{1}:\omega_{N_{x,y}})}{{\bar{v}}_{x,y}(\omega_{1}:\omega_{N_{x,y}})}\right)$$

where $\bar{H}$*_z_*(ω_1_:ω_Nz_), ${\bar{H}}_{x,y}$(ω_1_:ω_Nz_), $\bar{i}$_total,z_, $\bar{i}$_total,x,y_, $\bar{v}$*_z_*, and ${\bar{v}}_{x,y}$, respectively, represent the average sensitivity of the out-of-plane-sensing region, the average sensitivity of the in-plane-sensing regions, the average output current of the out-of-plane-sensing region, the average output current of the in-plane-sensing regions, the average arrived out-of-plane velocity of the out-of-plane-sensing region, and the average arrived in-plane velocity of the in-plane-sensing region, in the discrete range of [*ω*_1_, *ω*_*Nx*,*y*_]. In addition, *R_z_*, *R*_*x*,*y*_, *U_z_*, and *U*_*x*,*y*_, respectively, represent the transimpedance amplifier coefficient of the out-of-plane-sensing region, the transimpedance amplifier coefficient of the in-plane-sensing regions, the voltage amplifier coefficient of the out-of-plane-sensing region, and the voltage amplifier coefficient of the in-plane-sensing regions. Especially for the narrowband 3D-CMUT, *N*_*x*,*y*_ = *N_z_* = 1, and for broadband 3D-CMUT, *ω*_*Nx*,*y*_ = *ω_Nz_*.

For realizing the consistency in the average sensitivity, we should let Equation (3) be equal to Equation (4). Adjusting the coefficients of the amplification circuits, i.e., *R_z_*, *R*_*x*,*y*_, *U_z_*, and *U_z_*, may be an effective and convenient way to realize the consistency in the average sensitivity, because $\bar{i}$*_total_*_,*z*_ and $\bar{i}$*_total_*_,*x*,*y*_ are determined by several factors, e.g., the structural design, DC biased voltage, and cell number, and $\bar{v}$*_z_* and $\bar{v}$_*x*,*y*_ are independent on the 3D-CMUT.

### 2.2. A 3D-CMUT with Resonant Frequency of 200 kHz

In this section, we have chosen one unit of the 3D-CMUT as an example to illustrate its consistency in sensitivity. The design of the 3D-CMUT incorporates a piston-type CMUT cell (I) and two comb-like CMUT cells (II and III) that are all designed to resonate at a frequency of 200 kHz with a quality factor of 12.5. This ensures consistency in the sensitive frequency band. It is worth mentioning that 200 kHz was chosen as the central frequency because there are only low-order guided wave modes in the plate for the millimeter-thick plate structure [19], which helps us to verify the three-dimensional sensing performance of the 3D-CMUT device in subsequent studies.

**Figure 2:**
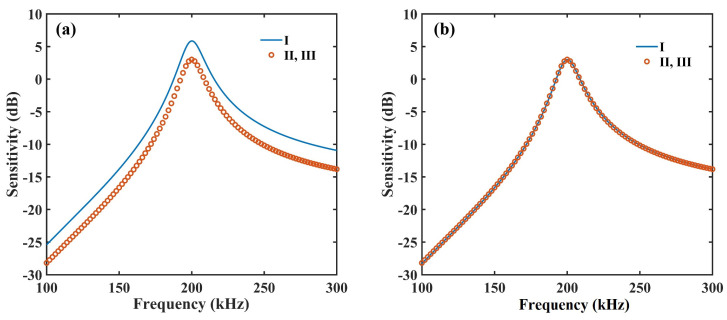
(**a**) Sensitivity of cell I, cell II, and cell III; (**b**) Unified sensitivity.

The structural parameters and material parameters for a 3D-CMUT with frequency of 200 kHz are shown in Table 1 and Table 2, respectively. According to the theoretical studies in Refs. [16,17], the frequency-dependent sensitivities of cell I, cell II, and cell III are depicted in Figure 2a, where, for cell I, the resonant frequency is 200 kHz, the amplification coefficient is *R_z_U_z_* = 10^5^ Ω, and the peak sensitivity is 5.8614 dB; for cell II and cell III, the resonant frequency is 200 kHz, the amplification coefficient is *R*_*x*,*y*_*U*_*x*,*y*_ = 10^5^ Ω, and the peak sensitivity is 3 dB. Based on the condition that Equation (3) is equal to Equation (4), we could obtain the relation of *R_z_U_z_*/*R*_*x*,*y*_*U*_*x*,*y*_ = 0.71957. Assuming the value of *R_z_U_z_* is 71,957 Ω, the value of *R*_*x*,*y*_*U*_*x*,*y*_ can be obtained as 10^5^ Ω correspondingly. Therefore, the consistency of average sensitivity can be realized by setting the amplification coefficients of the piston-type CMUT (cell I) and the comb-like CMUT (cell II and cell III) as *R_z_U_z_* = 71,957 Ω and *R*_*x*,*y*_*U*_*x*,*y*_ = 10^5^ Ω, respectively. Finally, Figure 2b shows the unified sensitivity, and the frequency-dependent sensitivities of cell I, cell II, and cell III show high consistencies.

## 3. Working Characteristics of 3D-CMUT by Finite Element Analysis

**Figure 3:**
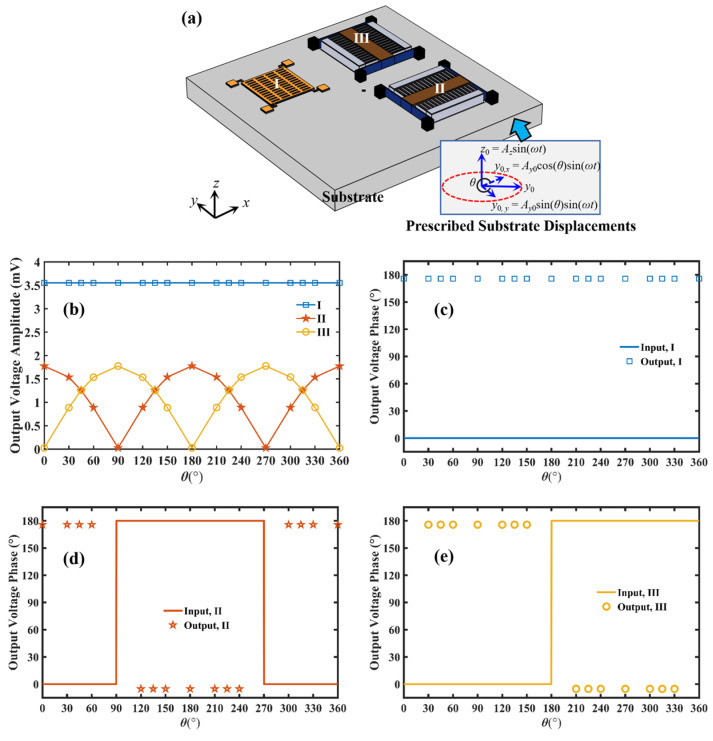
Performance of 3D-CMUT (**a**) Finite element simulation diagram of 3D-CMUT; (**b**) the output voltage amplitude of each cell changing with the direction of guided waves; (**c**) the phase of the input and the output signal of cell I, under different directions of guided waves; (**d**) the phase of the input and the output signal of cell II, under different directions of guided waves; (**e**) the phase of the input and the output signal of cell III, under different directions of guided waves.

In this section, a commercially available FEM modeling software, COMSOL Multiphysics, is adopted to simulate the working characteristics of the designed 3D-CMUT device with resonant frequency of 200 kHz, as shown in Figure 3a. The 3D-CMUT, which includes cells I, II and III, is attached to the surface of a plate. For a resonant 3D-CMUT, the simulation model is established by the “solid mechanics physics (*solid*)”, and the “electrostatics physics (*es*)” modules. In the “electrostatics physics (*es*)” module, both of the top and bottom electrodes of cell I (i.e., the piston-type CMUT cell), are considered as terminals. The top electrode is applied with a DC-biased voltage, and the bottom electrode is grounded. Similarly, both the stator and rotor of cell II (or cell III) are considered as terminals. The rotor is excited with a DC-biased voltage, and the stators are electrically grounded. Based on previous simulation experience, for the aim of analyzing guided waves accurately, the maximum size of the mesh should not be larger than one-sixth of the minimum wavelength. For the transient study in the next section, the time step performed by the solver should be set at least 1/(60 × *f*_m_), where *f*_m_ is the maximum frequency that needs to be solved.

In the frequency-domain simulation, we examine the output voltage responses of cell I, II and III of 3D-CMUT by exciting in-plane or out-of-plane harmonic waves at a specific frequency in the substrate. The expressions of prescribed substrate displacements are shown in Figure 3a. The out-of-plane displacement and in-plane displacement of the base are kept constant at *z*_0_ and *y*_0_, respectively, and expressed in terms of angle *θ*. Hereby, the excitation frequency is set at 200 kHz, which is equal to the resonance frequency of each cell. The in-plane displacement *y*_0_ is continuously adjusted from 0° to 360°, simulating the guided wave signals in different directions sensed by the 3D-CMUT. In addition, assuming the amplitudes of *z*_0_ and *y*_0_ are 2 nm and 1 nm, respectively, the output current of each element is converted to an amplified output voltage using integrated circuits.

Based on the characteristics of each sensing element, cell I is sensitive to out-of-plane displacement and used to sense the *z*_0_ component. Cell II is sensitive to in-plane displacement along the *x*-axis, and cell III is sensitive to in-plane displacement along the *y*-axis. Due to the small size of the CMUT element compared to the wavelength of the incident sound wave in the plate structure, the incident sound wave can be considered to act simultaneously on the three sets of CMUT cell structures, minimizing any phase difference introduced by small positional differences.

The output voltage amplitude of each element as a function of the direction angle *θ* is shown in Figure 3b. We can find that the output voltage amplitude of cell I remains constant, consistent with the constant amplitude of out-of-plane displacement. The output voltage amplitude of cell II exhibits a variation that matches the variation in the *y*_0_ component along the *x*-axis, while the output voltage amplitude of cell III exhibits a variation that matches the variation in the *y*_0_ component along the *y*-axis. The output voltage amplitude performance of each element reflects the unique characteristics of each element in the 3D-CMUT, with each element performing its designated task without interfering with others. The sensing sensitivity of each region is consistent.

Figure 3c–e depict the input displacement phase and output voltage phase of cells I, II, and III as a function of the direction angle *θ*. It should be noted that as the angle *θ* changes, the *y*_0_ component along the *x*-axis or *y*-axis may have negative values. For instance, at *θ* = 120°, the *y*_0_ component along the *x*-axis is −0.5 nm. When the input displacement is a positive real number, its phase angle is 0°. Conversely, when the input displacement is a negative real number, its phase angle is 180°. As the direction angle changes, the phase angle of the input displacement alternates between 0° and 180°. As a result, there is always a phase difference of 180° between the input displacement of each element and the phase angle of the output current. This indicates that the symbols (positive or negative) of the input and output of each element are opposite at the resonance frequency of 200 kHz. This phenomenon can be explained by the vibrations of each element. For example, when the out-of-plane displacement of cell I is positive (i.e., the out-of-plane displacement is along the positive *z*-axis), it causes the upper electrode of cell I to vibrate in the positive *z*-axis direction. This results in an increase in the air gap height and a decrease in capacitance, leading to a negative current.

By combining the amplitude and phase information in Figure 3b–e, the response of the 3D-CMUT to guided waves in different directions can be determined. Thus, it can be concluded that the 3D-CMUT exhibits excellent sensitivity to the direction of guided waves. This characteristic makes it valuable for applications in guided wave detection, such as acoustic emission detection in plates.

## 4. 3D-CMUT for Sensing Low-Order Guided Waves in Plate

In this section, the performance of 3D-CMUT in sensing transient guided waves is studied. It is well known that there are rich ultrasonic guided wave modes in plate structures. According to the dispersion curve of the guided wave in aluminum plate, when the frequency-and-thickness multiplication product (*fd*) is small, usually less than 1.5 MHz·mm, there are only three low-order guided wave modes in the plate, namely, the A_0_, S_0_ and SH_0_ modes [19]. For example, when the aluminum plate is 3 mm thick and the excitation frequency is 200 kHz, the *fd* value is 0.6 MHz·mm. Hence, there are only three low-order guided waves modes (A_0_, S_0_ and SH_0_) to be studied in a simulation environment such as COMSOL.

**Figure 4:**
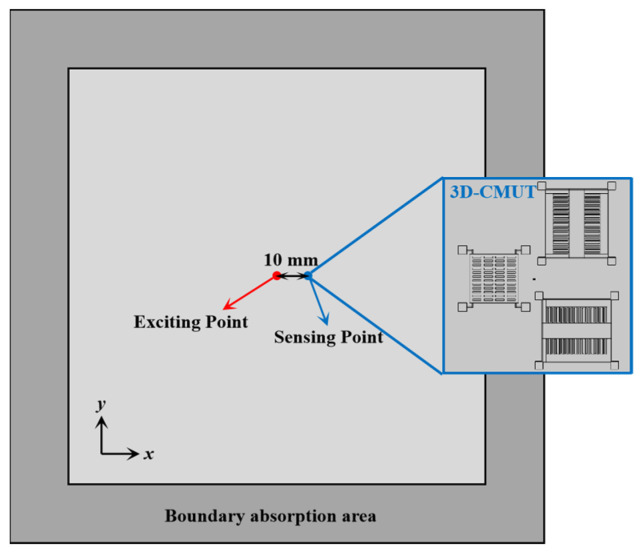
Schematic diagram of the design of sensing ultrasonic guided waves by 3D-CMUT.

Assume that a 3D-CMUT is attached to the surface of an aluminum plate that has a 3 mm thickness, as shown in Figure 4. A pair of excitation points are set at the center position of the aluminum plate to stimulate the A_0_, S_0_ and SH_0_ guided waves, where one excitation point is located at the center of the upper surface of the plate (as shown by the red point in Figure 4), and the other excitation point is symmetrically positioned on the lower surface of the plate. At the right side of the front excitation point is set a receiving (sensing) point. In addition, to reduce the influence of the boundary reflected waves, a boundary absorption area is set around the aluminum plate.

For the A_0_ wave excitation, the out-of-plane displacements of the two-point sources are identical. A Hanning-window tone burst A_0_ signal is generated, and the time-domain incident signal of the excitation points in the upper surface and the lower surface are expressed as [20,21]:

(5) displacementupper_surface_point(t)=z0(1−cos2πfctN)sin(2πfct)displacementlower_surface_point(t)=z0(1−cos2πfctN)sin(2πfct)

where *f*_c_ is the center frequency, *z*_0_ is the out-of-plane displacement amplitude, and *N* is the number of cycles.

For the S_0_ wave excitation, the out-of-plane displacements of the two-point sources are opposite. A Hanning-window tone burst S_0_ signal is generated, and the time-domain incident signal of the excitation points in the upper surface and the lower surface are expressed as:

(6) displacementupper_surface_point(t)=z0(1−cos2πfctN)sin(2πfct)displacementlower_surface_point(t)=−z0(1−cos2πfctN)sin(2πfct)

For the excitation of SH_0_ waves, the same in-plane displacement is loaded along the *y*-axis direction for the two-point source. A Hanning-window tone burst SH_0_ signal is generated along the *x*-axis direction, and the time-domain incident signal of the excitation points in the upper surface and the lower surface are expressed as:

(7) displacementupper_surface_point(t)=y0(1−cos2πfctN)sin(2πfct)displacementlower_surface_point(t)=y0(1−cos2πfctN)sin(2πfct)

where *y*_0_ is the in-plane displacement amplitude along the *y*-axis direction.

In calculations, *z*_0_ is 10 nm, *y*_0_ is 10 nm, *f*_c_ is 200 kHz, and *N* is 10. It should be pointed out that, in the simulation, when 3D-CMUT is excited with a DC-bias voltage at time zero, the 3D-CMUT cells will be forced to vibrate, and the vibration will disappear quickly within a duration of approximately 0.2 ms. To avoid the interference caused by the excited DC-bias voltage at time zero, the excitation time of the two-point source is set after 0.2 ms. For the excitation signal with a central frequency *f*_c_, we set the excitation time point of the guided waves at 50/*f*_c_ (here, 0.25 ms for *f*_c_ = 200 kHz).

**Figure 5:**
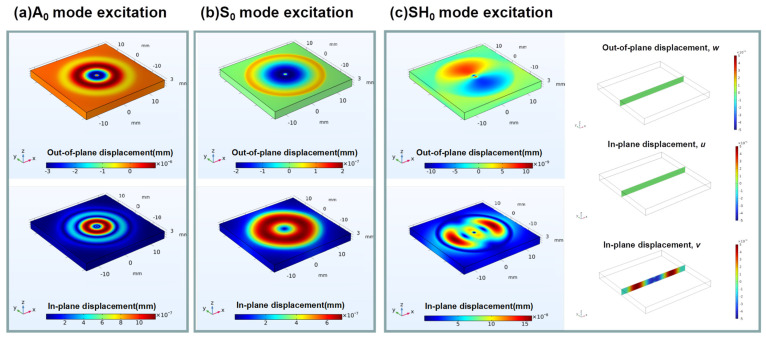
Excitation of A_0_, S_0_ and SH_0_ modes by two-point source method.

Figure 5 shows the guided wave displacement fields at a time of 0.28 ms after the A_0_, S_0_ and SH_0_ mode excitations. The out-of-plane displacement and the absolute in-plane displacement for A_0_, S_0_ and SH_0_ mode excitation are shown in Figure 5a, Figure 5b and Figure 5c, respectively. It should be pointed out that we can obtain the spherical extended A_0_ and S_0_ mode ultrasonic guided waves, but only obtain SH_0_ waves along the *x*-axis direction via the two-point source excitation method. We can also find that the A_0_ mode has dominant out-of-plane displacement, while the S_0_ mode has dominant in-plane displacement. For SH_0_ mode excitation, as shown in Figure 5c, the SH_0_ mode only exists in the *x*-o-*z* cross section. And the SH_0_ wave travels along the *x*-axis with a dominant in-plane displacement while the out-of-plane displacement is almost zero.

**Figure 6:**
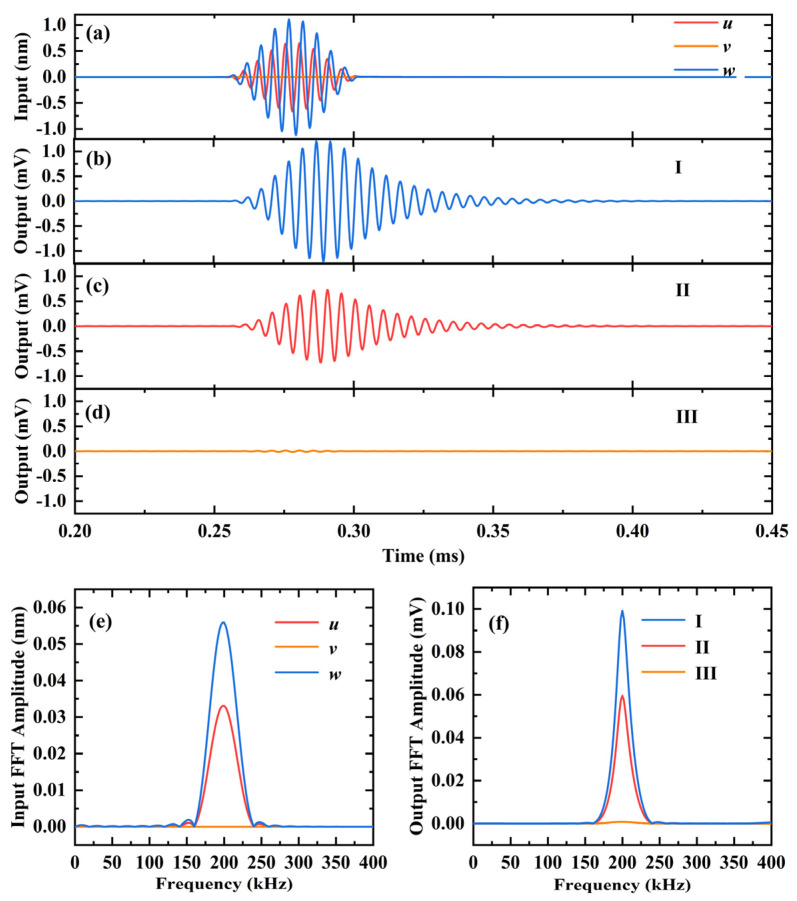
Finite element simulation results of using 3D-CMUT to sense the A_0_ Lamb wave with a center frequency of 200 kHz, in an aluminum plate with a thickness of 3mm. (**a**) The arrived in-plane displacements and out-of-plane displacements; (**b**) the output voltage of cell I; (**c**) the output voltage of cell II; (**d**) the output voltage of cell III; (**e**) FFT results of the arrived in-plane displacements and out-of-plane displacements; (**f**) FFT results of the output voltage of each cell.

The performance of the 3D-CMUT device in sensing A_0_ mode Lamb waves is illustrated in Figure 6. Figure 6a depicts the signals of out-of-plane displacement (*z*) and in-plane displacement (*x* or *y*) reaching the receiving point. Here, *u*, *v*, and *w* represent the signals in the *x*, *y*, and *z*-directions, respectively. The out-of-plane displacement (*z*) has an amplitude approximately 1.67 times that of the in-plane displacement (*x*). Since the receiving point is located directly to the right of the excitation point (*θ* = 0°), the in-plane displacement component along the *y*-axis is zero, resulting in the *v* signal reaching the receiving point being a straight line with a value of zero.

Figure 6b–d demonstrate the output results of A_0_ mode Lamb wave signals sensed by cells I, II, and III in 3D-CMUT. The output voltage amplitude of cell I is around 1.219 mV, cell II is approximately 0.7225 mV, and cell III is nearly 0 mV. The ratio of the output voltage amplitudes of cell I and II is 1.68, which is almost equal to the ratio of the out-of-plane displacement (*z*) amplitude of the Lamb wave signal reaching A_0_ mode to the in-plane displacement (*x*) amplitude. As the in-plane displacement (*y*) of the arrival signal is almost zero, cell III exhibits almost no voltage output. By utilizing fast Fourier transform (FFT), the input displacement spectrum and the output voltage spectrum of the 3D-CMUT device in the frequency domain can be obtained, as shown in Figure 6e and Figure 6f, respectively. It can be observed that the center frequency of the displacement signal reaching the receiving point, and the output voltage signal of each cell in the 3D-CMUT device, are both 200 kHz. Consequently, each cell successfully detects the A_0_ Lamb wave frequency of 200 kHz and its nearby guided wave three-dimensional signal.

**Figure 7:**
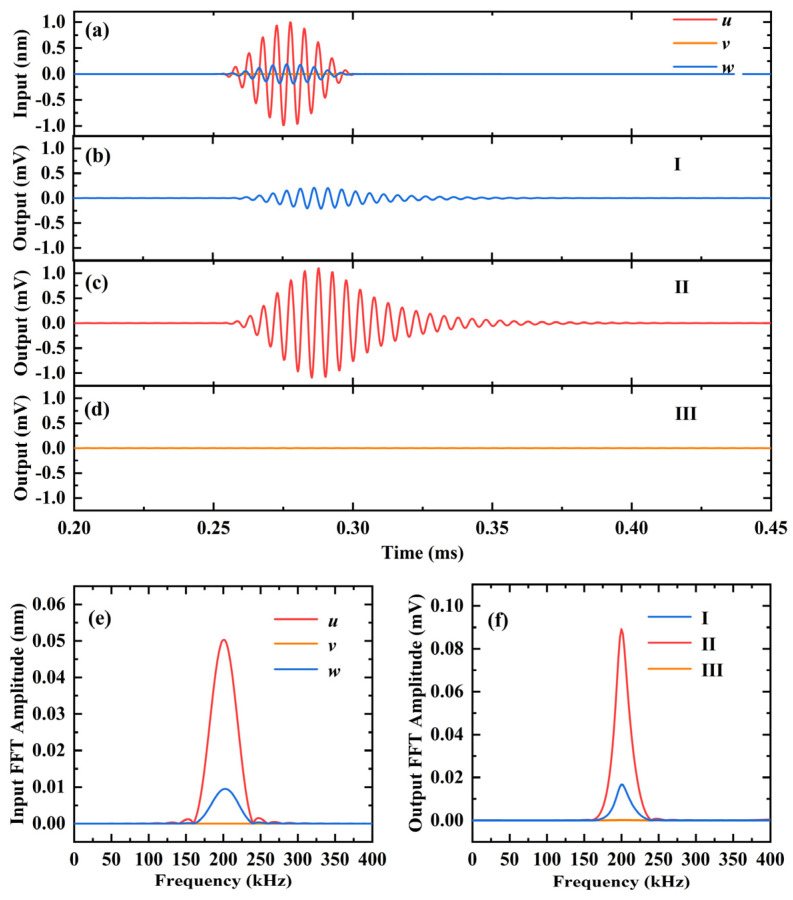
Finite element simulation results of using 3D-CMUT to sense the S_0_ Lamb wave with a center frequency of 200 kHz, in an aluminum plate with a thickness of 3 mm. (**a**) The arrived in-plane displacements and out-of-plane displacements; (**b**) the output voltage of cell I; (**c**) the output voltage of cell II; (**d**) the output voltage of cell III; (**e**) FFT results of the arrived in-plane displacements and out-of-plane displacements; (**f**) FFT results of the output voltage of each cell.

Figure 7 illustrates the performance of 3D-CMUT in receiving S_0_ mode Lamb waves. Figure 7a shows the signals of out-of-plane displacement (*z*) and in-plane displacement (*x* or *y*) reaching the receiving point. The amplitude of out-of-plane displacement (*z*) is 0.1887 nm, the amplitude of in-plane displacement (*x*) is 0.9893 nm, and the ratio of in-plane displacement (*x*) to out-of-plane displacement (*z*) amplitude is approximately 5.24. Similar to the A_0_ example, the *v* signal reaching the receiving point is a straight line with a zero value as a result of the receiving point being directly to the right of the excitation point (*θ* = 0°).

Figure 7b–d display the output results of S_0_ mode Lamb wave signals sensed by cells I, II, and III in 3D-CMUT, respectively. The output voltage amplitude of cell I is approximately 0.2087 mV, cell II is around 1.099 mV, and cell III is almost 0 mV. The ratio of output voltage amplitudes of cell II and I is 5.26, which is nearly equal to the ratio of in-plane displacement (*x*) to out-of-plane displacement (*z*) amplitude of the Lamb wave signal reaching S_0_ mode. Moreover, due to the in-plane displacement (*y*) of the arrival signal being nearly zero, cell III exhibits almost no voltage output.

The results of using the FFT to transform the displacement signal reaching the receiving point and the output voltage signal in the time domain of each cell in the 3D-CMUT into the frequency domain are depicted in Figure 7e and Figure 7f, respectively. It can be observed that the center frequency of the displacement signal reaching the receiving point and the output voltage signal of each cell in 3D-CMUT are both 200 kHz. Therefore, each cell successfully detects the guided wave 3D signal at and near the S_0_ Lamb wave frequency of 200 kHz.

**Figure 8:**
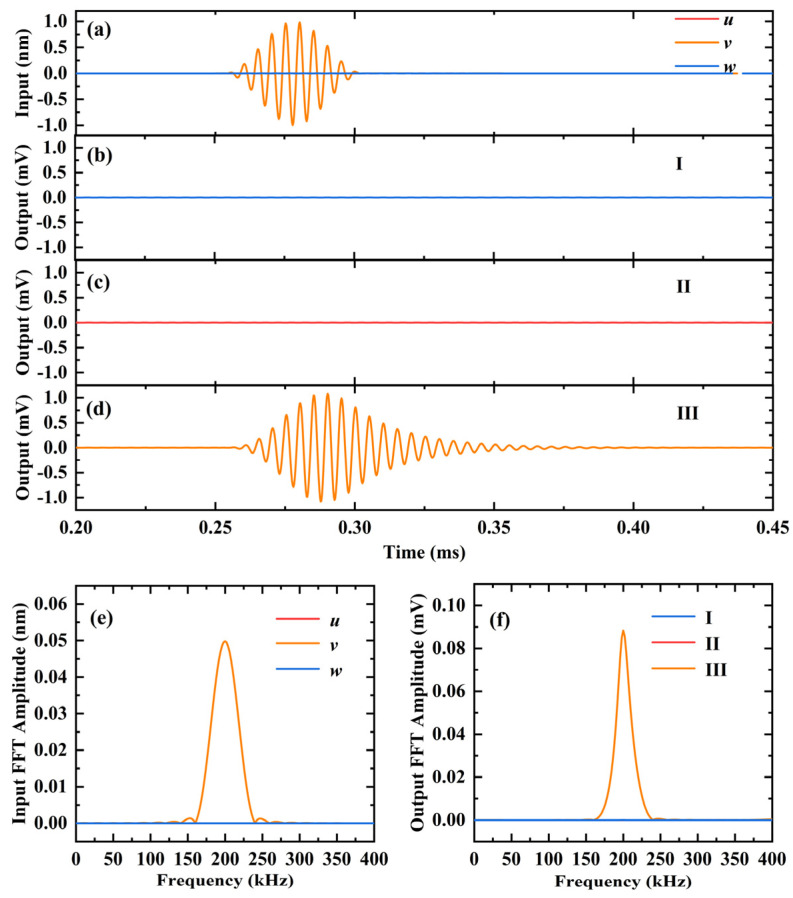
Finite element simulation results of using 3D-CMUT to sense the SH_0_ wave with a center frequency of 200 kHz, in an aluminum plate with a thickness of 3 mm. (**a**) The arrived in-plane displacements and out-of-plane displacements; (**b**) the output voltage of cell I; (**c**) the output voltage of cell II; (**d**) the output voltage of cell III; (**e**) FFT results of the arrived in-plane displacements and out-of-plane displacements; (**f**) FFT results of the output voltage of each cell.

The results of using 3D-CMUT to sense SH_0_ mode horizontal shear waves are shown in Figure 8. Figure 8a illustrates the out-of-plane displacement (*z*) and in-plane displacement (*x* or *y*) signals reaching the receiving point. Unlike A_0_ mode Lamb waves and S_0_ mode Lamb waves, the horizontal shear waves in SH_0_ mode exhibit only in-plane displacement. The displacement of the excitation point along the *y*-direction results in in-plane displacement reaching the receiving point with only *y*-direction components. At the receiving point, the amplitudes of out-of-plane displacement (*z*) and in-plane displacement (*x*) are both 0 nm, while the amplitude of in-plane displacement (*y*) is 0.9960 nm.

Figure 8b–d show the output results of SH_0_ mode horizontal shear wave signals received by the sensors of cells I, II, and III in the 3D-CMUT, respectively. Among them, the output voltage amplitudes of cells I and II are almost 0 mV, while the output voltage amplitude of cell III is approximately 1.084 mV. The results of applying FFT to transform the displacement signal in the time domain and the output voltage signal in the time domain of each cell in the 3D-CMUT into the frequency domain are shown in Figure 8e and Figure 8f, respectively. It can be observed that among the displacement signals reaching the receiving point and the output voltage signals of each cell in the 3D-CMUT, only the in-plane displacement (*y*) and the spectrum of cell III are non-zero, with a center frequency of 200 kHz. Each cell successfully detected the SH_0_ mode horizontal shear wave frequency of 200 kHz and its nearby guided wave three-dimensional signals.

## 5. 3D-CMUT for Acoustic Emission (AE) Detection in Plate Structures

This section aims to explore the potential application of the 3D-CMUT device in the field of acoustic emission detection. The focus is on investigating the feasibility of using 3D-CMUT to detect acoustic emission signals by simulating the excitation of a section of an acoustic emission signal in a thin plate and receiving it using the 3D-CMUT.

**Figure 9:**
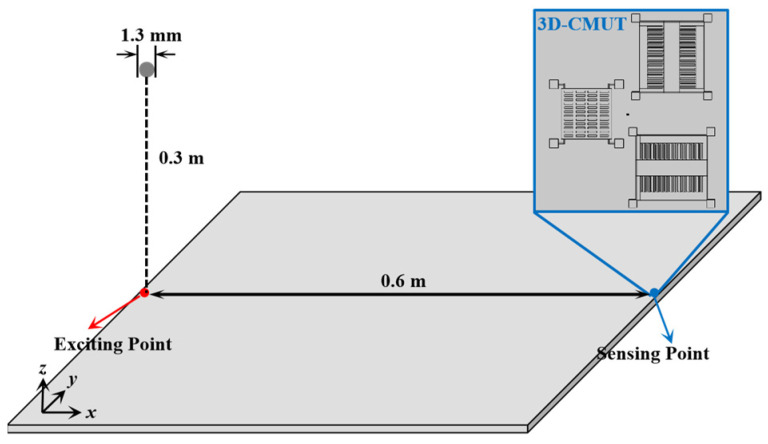
Schematic diagram of the design of detecting the acoustic emission signal from ball drop impact by using the 3D-CMUT sensor.

Figure 9 shows the schematic diagram of the design for detecting the acoustic emission signal from a ball drop impact by using the 3D-CMUT sensor. In the finite element transient simulation, a theoretical equivalent force is utilized to represent the impact of a falling ball on a plate, which will produce acoustic emission signals in the plate. The force generated on the specimen surface by a free-falling ball can be approximated using the following expression [22]:

(8) $$F(t)=\frac{2.28m_{0}gh}{\alpha }\sin\left(\frac{1.51\sqrt{gh}}{\alpha }(t-t_{0})\right),\;t_{0}\leq t\leq (t_{0}+\pi \alpha /1.51\sqrt{gh})$$

where,

$$\alpha ={\left(\frac{15\pi (\delta_{0}+\delta_{1})m_{0}gh}{8\sqrt{R_{0}}}\right)}^{0.4}$$

$$\delta_{0}=(1-\upsilon_{0})/(\pi E_{0})$$

$$\delta_{1}=(1-\upsilon_{1})/(\pi E_{1})$$

and *m*_0_ represents the mass of the small ball, *R*_0_ represents the diameter of the small ball, $\upsilon_{0}$ is the Poisson ratio of the material of the ball, *E*_0_ represents the Young’s modulus of the small ball material, $\upsilon_{1}$ represents the Poisson ratio of the specimen material, *E*_1_ represents the Young’s modulus of the specimen material, *g* is the gravitational acceleration, *h* is the height of the free-drop distance from the specimen surface, and *t*_0_ is the time point when the small ball is just in contact with the specimen surface. Specifically, the AE signal-excitation time is 50/*f*_c_, where *f*_c_ is the resonance frequency of the designed 3D-CMUT device, the height of the small ball free fall is 0.3 m, the radius of the pellet is 1.3 mm, the thickness of the sheet is 3 mm, and the materials of the ball and the sheet are steel and aluminum, respectively.Furthermore, the 3D-CMUT device was placed to the right side of the excitation point at a distance of 0.6 m.

**Figure 10:**
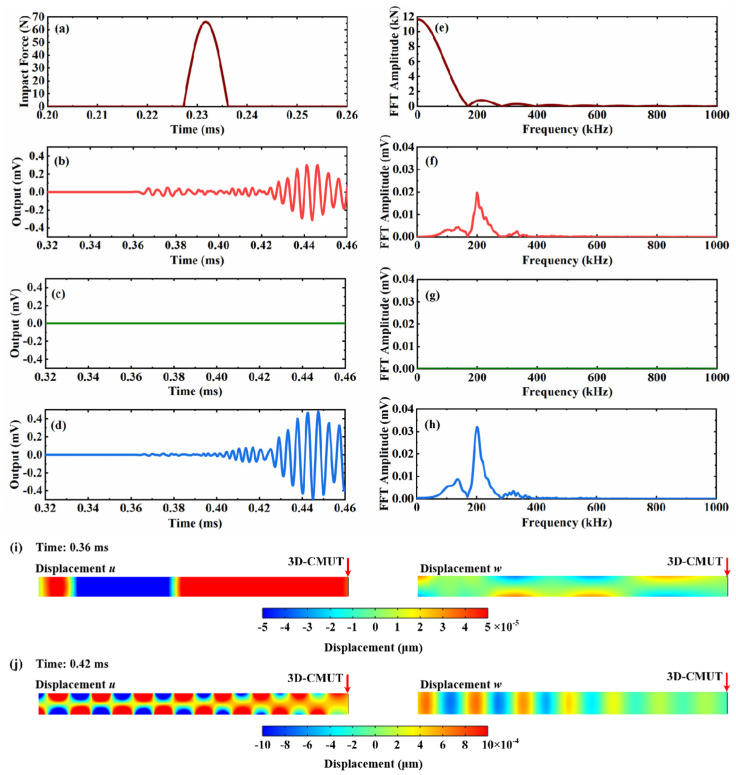
Results of detecting the acoustic emission signal from ball drop impact by 3D-CMUT. (**a**) The time-domain signal of the ball drop impact; (**b**) the output voltage of cell II; (**c**) the output voltage of cell III; (**d**) the output voltage of cell I; (**e**) FFT results of (**a**); (**f**) FFT results of (**b**); (**g**) FFT results of (**c**); (**h**) FFT results of (**d**); (**i**) the out-of-plane (*w*) and the in-plane (*u*) displacement distributions of the plate at 0.36 ms; (**j**) the out-of-plane (*w*) and the in-plane (*u*) displacement distributions of the plate at 0.42 ms.

Figure 10 presents the simulation results of the acoustic emission signal generated by the impact of a small ball falling on a thin plate using the detecting capability of the 3D-CMUT device. At 0.25 ms, when the impact force is applied to the thin plate, the maximum force value is approximately 66 N, and the force spectrum covers a frequency range of approximately 0 kHz to 400 kHz.

Figure 10b–d show the output voltages of cells I, II, and III in the 3D-CMUT. Cells I and II sense the out-of-plane displacement (*w*) and in-plane displacement (*u*) components of the acoustic emission signal, respectively. Because of the absence of in-plane displacement (*v*) components in the acoustic emission signal, the output voltage of cell III is always zero.

At approximately 0.361 ms, the first acoustic emission signal reaches the 3D-CMUT device. By examining the displacement field components *u* and *w* in the cross-section of the thin plate shown in Figure 10i, it can be observed that the first signal in the cross-section exhibits a symmetric distribution of the displacement field *u* along the thickness direction, while the displacement field *w* shows an antisymmetric distribution along the thickness direction. Considering that the propagation speed of the first segment of the signal is approximately 5405 m/s, similar to the group velocity of 5254 m/s of the 200 kHz S_0_ Lamb wave, the first segment of the signal can be identified as the S_0_ Lamb wave. However, due to the short propagation distance, some A_0_ Lamb wave signals overlap with S_0_ Lamb wave signals, making it challenging to accurately determine the arrival time of A_0_ Lamb waves. Nevertheless, as depicted in Figure 10j, at approximately 0.42 ms, the signal demonstrates an antisymmetric distribution of the displacement field *u* along the thickness direction and a symmetric distribution of the displacement field *w* along the thickness direction. At this point, the A_0_ Lamb wave has reached the 3D-CMUT device. Additionally, from the frequency spectrum of the voltage signal shown in Figure 10f–h, it is evident that the 3D-CMUT successfully detected an acoustic emission signal with a frequency of approximately 200 kHz.

To determine the location of acoustic emission sources, location theories such as the triangulation method and four-point arc method [23,24,25] can be employed. Taking the triangulation method as an example, using the propagation speed and arrival time of the S_0_ Lamb wave, a circle centered at the 3D-CMUT device can be determined, representing the possible position of the acoustic emission source. Using three 3D-CMUT devices attached to different positions, three circles can be determined, and the intersection of these circles is the location of the evaluated acoustic emission source.

It is important to note the dominant nature of in-plane displacement (*u*) in the S_0_ Lamb wave generated by the acoustic emission source. Cell I can only detect its very small out-of-plane displacement (*w*) signal, which is easily masked by noise signals. On the other hand, cell II can detect the larger in-plane displacement (*u*) signal, resulting in a stronger S_0_ Lamb wave signal output. In contrast to piezoelectric acoustic emission sensors that focus solely on single-dimensional information sensing, the 3D-CMUT device achieves highly sensitive sensing of the three-dimensional information of the acoustic emission signal at the same test point. This is accomplished through the combination of out-of-plane displacement-sensing CMUT cells and in-plane displacement-sensing CMUT cells. As a result, it is difficult to lose crucial information, highlighting the value of the 3D-CMUT device in acoustic emission detection.

## 6. Conclusions

In this study, a novel ultrasonic guided wave sensor called the 3D-CMUT was designed, which combines an out-of-plane displacement-sensing CMUT cell and an in-plane displacement-sensing CMUT cell. The sensor enables cooperative sensing of the in-plane and out-of-plane displacement of ultrasonic guided waves in a small area of a plate structure. Finite element simulations were conducted, and the key findings are summarized as follows:

A method was proposed to achieve uniform sensing sensitivity in the *x*, *y*, and z-directions for a narrowband and broadband 3D-CMUT. This method is based on the design of transimpedance amplifiers, providing higher flexibility and stability.

To simplify the analysis, the 3D-CMUT was simplified to include one out-of-plane displacement-sensing CMUT cell and two orthogonally placed in-plane displacement-sensing CMUT cells. The working characteristics of the simplified 3D-CMUT were analyzed. The results of output amplitude and phase demonstrated that the 3D-CMUT is highly sensitive to the direction of guided waves. The application of the 3D-CMUT for sensing A_0_, S_0_, and SH_0_ waves revealed its excellent capability for three-dimensional information-sensing of guided waves.

The application of the 3D-CMUT in acoustic emission detection was demonstrated using the example of detecting acoustic emission signals generated by a free-falling steel ball hitting a thin aluminum plate. The results showed that the 3D-CMUT effectively senses A_0_ and S_0_ waves in acoustic emission signals. Particularly when there is a significant difference in the amplitude of out-of-plane displacement and in-plane displacement in a guided wave mode, the 3D-CMUT can still detect the displacement information in the respective directions. This highlights the versatility and accuracy of 3D-CMUT in capturing important information from acoustic emission signals.

Overall, this study presents a significant advancement in ultrasonic guided wave sensing through the development of the 3D-CMUT sensor. Its ability to simultaneously detect in-plane and out-of-plane displacement provides a comprehensive understanding of the behavior of ultrasonic guided waves in plate structures. This sensor has potential applications in various industries such as structural health monitoring, non-destructive testing, and acoustic emission detection. Further research and development of 3D-CMUT technology could lead to even more advanced and effective sensing capabilities in the field of ultrasonic guided wave sensing.

## Figures and Tables

**Table 1:** Specific structural parameters of the designed 3D-CMUT.

Piston-Type CMUT (Unit: μm)				Comb-Like CMUT (Unit: μm)			
*d_Au_*	0.5	*l_s_*_1_	11	*t_s_*	2	*d*_3_	4
*d_top_*	2	*w*	2.5	*l_s_*	54.28	*d*_4_	2
*d_gap_*	1.1	*a_h_*	20	*t_m_*	162	*t_f_*	2
$d_{Si_{3}N_{4}}$	0.35	*b_h_*	4	*l_m_*	36	*l*_1_	21
*d_bottom_*	0.7	*d*	5.7	*d*_1_	2	*l*_2_	33
*l_s_*_1_	14.5	*l*	114	*d*_2_	2		

**Table 2:** Material parameters employed in the designed 3D-CMUT.

	Polycrystalline Silicon	Gold	Si_3_N_4_
Mass density (kg/m^3^)	2320	19,300	3100
Young’s modulus (GPa)	160	70	250
Poisson’s ratio	0.22	0.44	0.23
Relative permittivity	4.5	--	9.7

## Data Availability

Not applicable.

## References

[1] Mitra M., Gopalakrishnan S. (2016). Guided wave based structural health monitoring: A review. Smart Mater. Struct..

[2] Olisa S.C., Khan M.A., Starr A. (2021). Review of Current Guided Wave Ultrasonic Testing (GWUT) Limitations and Future Directions. Sensors.

[3] Driss H., El Mahi A., Bentahar M., Beyaoui M., Haddar M. (2023). Characterization of Tensile and Fatigue Damages in Composite Structures Using Lamb Wave for Improved Structural Health Monitoring. Int. J. Appl. Mech..

[4] Li P., Shan S., Wen F., Cheng L. (2018). A fully-coupled dynamic model for the fundamental shear horizontal wave generation in a PZT activated SHM system. Mech. Syst. Signal Process..

[5] Wu J., Zhang W.W. (2013). Circumference Damage Identification in a Pipe Using Mode Conversion of Longitudinal Guided Wave. Appl. Mech. Mater..

[6] He J., Leckey CA C., Leser P.E., Leser W.P. (2019). Multi-mode reverse time migration damage imaging using ultrasonic guided waves. Ultrasonics.

[7] Yu L., Santoni-Bottai G., Xu B., Liu W., Giurgiutiu V. (2010). Piezoelectric wafer active sensors for in situ ultrasonic-guided wave SHM. Fatigue Fract. Eng. Mater. Struct..

[8] Bua-Nunez I., Posada-Roman J.E., Rubio-Serrano J., Garcia-Souto J.A. (2014). Instrumentation System for Location of Partial Discharges Using Acoustic Detection with Piezoelectric Transducers and Optical Fiber Sensors. IEEE Trans. Instrum. Meas..

[9] Ge H., Huat D.C.K., Koh C.G., Dai G., Yu Y. (2022). Guided wave–based rail flaw detection technologies: State-of-the-art review. Struct. Health Monit..

[10] Chen D.M., Zhu W.D. (2021). Investigation of three-dimensional vibration measurement by three scanning laser Doppler vibrometers in a continuously and synchronously scanning mode. J. Sound Vib..

[11] Brenner K., Ergun A.S., Firouzi K., Rasmussen M.F., Stedman Q., Khuri-Yakub B.P. (2019). Advances in Capacitive Micromachined Ultrasonic Transducers. Micromachines.

[12] Bawiec C., N’Djin W., Bouchoux G., Sénégond N., Guillen N., Chapelon J.-Y. (2018). Preliminary Investigation of a 64-element Capacitive Micromachined Ultrasound Transducer (CMUT) Annular Array Designed for High Intensity Focused Ultrasound (HIFU). Innov. Res. Biomed. Eng..

[13] Ozevin D., Greve D.W., Oppenheim I.J., Pessiki S.P. (2006). Resonant capacitive MEMS acoustic emission transducers. Smart Mater. Struct..

[14] Saboonchi H., Ozevin D. (2013). MEMS acoustic emission transducers designed with high aspect ratio geometry. Smart Mater. Struct..

[15] Kabir M., Saboonchi H., Ozevin D. The design, characterization, and comparison of MEMS comb-drive acoustic emission transducers with the principles of area-change and gapchange. Proceedings of the Sensors and Smart Structures Technologies for Civil, Mechanical, and Aerospace Systems 2015.

[16] Lu W., Zhang S., Wang R., Xu B., Yilmaz M., Zhang W. (2022). Theoretical and Simulation Studies on Designing a Phase-Reversal-Based Broadband CMUT With Flat Passband and Improved Noise Rejections for SHM. IEEE Sens. J..

[17] Zhang S., Lu W., Yang Y., Wang R., Zhang G., Xu B., Yilmaz M., Zhang W. (2023). In-Plane-Sensing Analysis of Comb-Like Capacitive Micro-Machined Ultrasonic Transducers (CMUTs) Using Analytical Small-Signal Model and FEM. IEEE Sens. J..

[18] Capineri L., Bulletti A. (2021). Ultrasonic Guided-Waves Sensors and Integrated Structural Health Monitoring Systems for Impact Detection and Localization: A Review. Sensors.

[19] Rose J.L. (2014). Ultrasonic Guided Waves in Solid Media.

[20] Lu W., Zhang S., Wang R., Yang Y., Zhang G., Zhang W., Xu B., Yilmaz M. (2022). FEM-based analysis on sensing out-of-plane displacements of low-order Lamb wave modes by CMUTs. J. Appl. Phys..

[21] Niu X., Duan W., Chen H.-P., Marques H. (2019). Marques Excitation and propagation of torsional T(0,1) mode for guided wave testing of pipeline integrity. Measurement.

[22] Goldsmith W. (2001). Impact: The Theory and Physical Behavior of Colliding Solids.

[23] Aljets D., Chong A., Wilcox S., Holford K. (2012). Acoustic emission source location on large plate-like structures using a local triangular sensor array. Mech. Syst. Signal Process..

[24] Baxter M.G., Pullin R., Holford K.M., Evans S.L. (2007). Delta T source location for acoustic emission. Mech. Syst. Signal Process..

[25] Tobias A. (1976). Acoustic-emission source location in two dimensions by an array of three sensors. Non-Destr. Test..

